# Genome-wide identification and expression pattern analysis of lipoxygenase gene family in turnip (*Brassica rapa* L. subsp. *rapa*)

**DOI:** 10.7717/peerj.13746

**Published:** 2022-07-22

**Authors:** Cunyao Yan, Kai Jia, Jing Zhang, Zhonglin Xiao, Xiaomei Sha, Jie Gao, Huizhuan Yan

**Affiliations:** College of Horticulture, Xinjiang Agricultural University, Urumqi, Xinjiang, China

**Keywords:** Turnip, Lipoxygenase, Phylogenetic analysis, Gene expression, Abiotic stress

## Abstract

Turnip (*Brassica rapa* L. subsp.* rapa*) is an important crop with edible and medicinal values, and various stresses, especially salt stress and drought stress, seriously threaten the yield of turnips. LOXs play important roles in regulating plant growth and development, signal transduction, and biotic and abiotic stress responses through secondary metabolites produced by the oxylipin metabolic pathway, and although the turnip genome has been published, however, the role of *LOX* family genes in various abiotic stress responses has not been systematically studied in turnips. In this study, a total of 15 *LOX* genes (*BrrLOX*) were identified in turnip, distributed on six chromosomes. Phylogenetic tree analysis classified these *LOX* genes into two classes: three 9-LOX proteins and 12 13-LOX type II proteins. Gene duplication analysis showed that tandem and segmental duplication were the main pathways for the expansion of the *BrrLOX* gene family. The Ka and Ks values of the duplicated genes indicate that the *BrrLOX* gene underwent strong purifying selection. Further analysis of the cis-acting elements of the promoters suggested that the expression of the *BrrLOX* gene may be influenced by stress and phytohormones. Transcriptome data analysis showed that 13 *BrrLOX* genes were expressed at one or more stages of turnip tuber development, suggesting that *LOX* genes may be involved in the formation of turnip fleshy roots. The qRT-PCR analysis showed that four stresses (salt stress, drought stress, cold stress, and heat stress) and three hormone treatments (methyl jasmonate, salicylic acid, and abscisic acid) affected the expression levels of *BrrLOX* genes and that different *BrrLOX* genes responded differently to these stresses. In addition, weighted gene co-expression network analysis (WGCNA) of *BrrLOX* revealed seven co-expression modules, and the genes in these co-expression modules are collectively involved in plant growth and development and stress response processes. Thus, our results provide valuable information for the functional identification and regulatory mechanisms of *BrrLOX* in turnip growth and development and stress response.

## Introduction

Lipoxygenases (LOXs; EC 1.13.11.12) are non-heme iron-containing dioxygenases capable of generating fatty acid hydroperoxides by specifically catalyzing the oxygenation of polyunsaturated fatty acids (PUFAs) with a (1Z, 4Z)-pentadiene system (Feussner & Wasternack, 2002). These enzymes play important roles in plant growth, development, and stress response (Wasternack & Hause, 2013). Typically, LOXs are divided into two main categories depending on the oxygenation site on the PUFA chain: 9-LOX and 13-LOX, (oxygenation at the 9th or 13th carbon). Here, 9-LOX catalyzes the production of 9-HPOT (9-hydroperoxy-(10E,12Z)-octadecatrienoic acid), involved in stress (Ul Hassan, Zainal & Ismail, 2015), while 13-LOX catalyzes the production of 13-HP?T (13-hydroperoxy-(9Z,12E)-octadecatrienoic acid), which mediates the synthesis of jasmonic acid derivatives and participates in various life processes in various plants (Viswanath et al., 2020). The LOXs contain two structural domains, a conserved PLAT/LH2 (polycystin-1, lipoxygenase, alpha-toxin/lipoxygenase homology) structural domain at the N-terminal end and a highly conserved LOX structural domain at the C-terminal end. The PLAT/LH2 structural domain plays an important role in membrane binding. The LOX structural domain contains a highly conserved [His-(X)4-His-(X)4-His-(X)17-His-(X)8-His] region, which is rich in histidine (His) residues and plays an important role in the binding of LOX to iron and in exerting enzymatic activity (Andreou & Feussner, 2009; Steczko et al., 1992).

Many LOX genes have been cloned and functionally characterized, for example, in *Arabidopsis thaliana*, 6 *AtLOX* genes have been identified. *AtLOX1* and *AtLOX5* have been observed to be involved in lateral root development (Vellosillo et al., 2007). *AtL0X2*, *AtL0X3*, *AtLOX4* and *AtLOX6* are involved in JA synthesis, among them *AtLOX2* can be induced by the damage response (Bell, Creelman & Mullet, 1995), and *AtLOX6* is involved in crustacean defence and drought stress (Grebner et al., 2013), and *AtLOX3* and *AtLOX4* can regulate flower development (Caldelari et al., 2011). The ectopic expression of the *DkLOX3* gene of persimmon in tomato promoted ripening (Hou et al., 2015) and senescence, while the *CsLOX1* gene of cucumber in Arabidopsis regulated stamen development (Yong et al., 2020). The *CaLOX1* gene of Capsicum annuum ectopic expression in Arabidopsis enhances the tolerance of transgenic plants to high salt stress and severe drought stress (Lim et al., 2015). The ectopic expression of the oriental melon *CmLOX10* gene in Arabidopsis enhanced drought tolerance (Xing et al., 2020). These studies suggest that *LOX* genes play an important role in plant growth and development and in diverse stresses. Although LOX family genes have been identified in several plants such as radish (Wang et al., 2019), pepper (Sarde et al., 2018), poplar (Chen et al., 2015), watermelon (Liu et al., 2020), and pear (Li et al., 2014), however, no systematic analysis of the LOX family in turnips has been reported so far. With the completion of whole genome sequencing of turnips, it has become possible to identify and characterise the LOX family of genes in turnips.

Turnip (*Brassica rapa* L. subsp. *rapa*) is a biennial herb of the cruciferous family. They are important root vegetables in China and are grown mainly in Xinjiang and Qinghai (Paul et al., 2019). The Xinjiang region, however, is located in a relatively arid geographical environment with saline soils, and turnip seedlings are often affected by drought, salinity and other stresses during growth, resulting in reduced turnip yields (Jia et al., 2020). Therefore, in-depth studies on genes related to turnip stress signaling are important to improve stress resistance, reduce stress impact, and ultimately increase yield.

The study identified 15 members of the turnip LOX gene family based on available genomic information and analyzed the structure and evolution of *BrrLOXs* through phylogenetic analysis, gene structure, conserved structural domains, and gene duplication events. In addition, the expression patterns of the *BrrLOX* genes in the roots at different developmental stages were analyzed based on publicly available RNA-seq data. Further, the response of the *BrrLOX* genes to abiotic stresses was determined by qRT-PCR analysis. Genome-wide characterisation, evolutionary and expression pattern analysis of turnip LOX genes could provide the basis for future gene function studies and new ideas for improving turnip stress resistance.

## Materials and Methods

### Identification of the turnip *LOX* genes

The genome, CDS, and protein sequence files of turnip were downloaded from the Brassicaceae database (http://brassicadb.cn/#/) (Chen et al., 2021). The Hidden Markov Model file for lipoxygenase (PF00305) was downloaded from the Pfam database (https://pfam.xfam.org/) (Mistry et al., 2020), and the turnip genome was searched using HMMER 3.0 software and candidate LOX proteins with an e value < 1.0E−05 were screened (Wheeler & Eddy, 2013) and submitted to the conserved domains database (CDD, https://www.ncbi.nlm.nih.gov/cdd/?term=) to confirm the presence of lipoxygenase and the presence of PLAT/LH2 structural domain (Lu et al., 2020). After excluding sequences without the lipoxygenase and PLAT/LH2 structural domains, the non-redundant sequences were named according to their chromosomal position order. ExPASy (https://web.expasy.org/protparam/) was used to analyze the basic physicochemical properties of LOX proteins. The chloroplast transit peptides and the subcellular localization were predicted using TargetP 2.0 Server (https://services.healthtech.dtu.dk/service.php?TargetP-2.0) and WoLF PSORT (https://psort.hgc.jp/), respectively. Global sequence alignment was performed using the EMBOSS Needle tool (https://www.ebi.ac.uk/Tools/psa/emboss_needle/) to determine the similarity and identity between the *LOX* members. The conserved motifs of *LOX* (the number of motifs is set to 20) were analyzed using the MEME suite (https://meme-suite.org/meme/tools/meme) (Bailey et al., 2015), and the gene structure was mapped using TBtools software (Chen et al., 2020).

### Phylogenetic analysis

The published LOX protein sequences of Arabidopsis (Umate, 2011), radish (Wang et al., 2019), watermelon (Liu et al., 2020), cotton (Shaban et al., 2018), sorghum (Shrestha, Pant & Huang, 2021), buckwheat (Ji et al., 2020), banana (Liu et al., 2021), and poplar (Chen et al., 2015) were retrieved from (https://www.arabidopsis.org/), BRAD (http://brassicadb.cn/#/), CuGenDB (http://cucurbitgenomics. org/organism/1), CottonGen (https://www.cottongen.org/), NCBI (https://www.ncbi.nlm.nih.gov/), MBKBASE (http://www.mbkbase.org/Pinku1/), Banana Genome Hub (https://banana-genome-hub.southgreen.fr), and Phytozome (http://www.Phytozome.net/), respectively. Multiple sequence alignment was performed using MUSCLE software (Edgar, 2021) and phylogenetic trees were constructed by the neighbor-joining method using MEGA X software (Partial deletion; 95% site coverage cutoff, and 1,000 bootstrap replicates) (Kumar et al., 2018) and visualized using iTOL (https://itol.embl.de/) (Letunic & Bork, 2021).

### Chromosome localization and gene duplication analysis

Whole-genome duplication and tandem repeat relationships between *BrrLOX* genes and genetic covariance between species were detected using MCScanX software (https://github.com/wyp1125/MCScanX) (Wang et al., 2012). Chromosome location was obtained from the gene feature format (GFF) gene annotation files and Visualized gene co-linear relationships using Circos software (http://circos.ca) (Krzywinski et al., 2009). Then, the synonymous substitution rate (Ka) and the non-synonymous substitution rate (Ks) were calculated for gene pairs using KaKs_Calculator 2.0 software (Wang et al., 2010).

### Analysis of cis-acting elements in the promoters of turnip *LOXs*

A 2,000 bp sequence upstream of the start codon of each turnip *LOX* gene was extracted from the turnip genome database, and the cis-acting elements of the promoter were predicted using PlantCARE (http://bioinformatics.psb.ugent.be/webtools/plantcare/html/) (Lescot et al., 2002).

### Expression analysis of RNA-seq data from turnip fleshy roots

The raw transcriptome data of the three developmental periods (each with two independent biological replicates) of turnip fleshy roots were downloaded from NCBI (https://www.ncbi.nlm.nih.gov/bioproject/PRJNA273340/) (Li et al., 2015), the transcriptome data were remapped to the turnip reference genome using HISAT2 (Pertea et al., 2016), and the TPM values (Transcripts Per Kilobase of exon model per Million mapped reads) of turnip *LOX* were calculated using the featureCounts tool (Liao, Smyth & Shi, 2014), and finally the TPM values were normalized by a logarithmic scale with base 2 and visualized in a heat map using the TBtools software (Chen et al., 2020).

### Plant materials

We performed a transcriptome analysis of ‘Qiamagu’ turnip seedlings showing the transcriptome changes caused by four treatments (mock control; 100 µM MeJA spray; 100 mM NaCl watering; 100 µM MeJA spray + 100 mM NaCl watering) on leaves of four-leaf stage seedlings (Table S1), with three biological replicates of each treatment. The plant material used for the stress and hormone treatments was the Xinjiang cultivar ‘Qiamagu’. Turnips were grown at 25 °C in a light incubator with 16 h light/8 h dark photoperiod and 60% relative humidity. Seedlings of uniform size and two weeks old were selected for stress and hormone treatments. To examine the effect of methyl jasmonate (MeJA), salicylic acid (SA), and abscisic acid (ABA) treatments on turnip *LOX*, leaves were sampled at 0, 4, 8, 12, and 24 h after spraying 100 µM of MeJA, SA, and ABA, respectively. To investigate the effect of abiotic stress on turnip *LOX*, turnip plants at the four-leaf stage were watered with 100 mM NaCl and 20% PEG6000 to simulate salt stress and drought stress, respectively, and exposed to 4 °C and 40 °C to simulate low and high temperatures, respectively. The leaves were sampled at 0, 4, 8, 12, and 24 h after treatment, immediately frozen in liquid nitrogen, and stored at −80 °C for further use.

### Real-time quantitative PCR

Total RNA was extracted from the leaf samples using RNAprep pure Plant Kit (Tiangen, Beijing, China) and reverse-transcribed to generate the first-strand cDNA using RT reagent Kit with gDNA Eraser (Takara, Beijing, China). The cDNA was diluted and used for qRT-PCR. Primers for BrrLOX family genes used in qRT-PCR were designed using Primer-BLAST (https://www.ncbi.nlm.nih.gov/tools/primer-blast/index.cgi) with turnip *β*-Actin as the internal reference gene (Ye et al., 2012). All primers used in this study are listed in Table S2. Relative expression levels of the *BrrLOX* genes were calculated following the 2^−ΔΔCt^ method. Three biological replicates were set up for each time point per treatment, with each repetition consisting of nine plants.

### Weighted gene co-expression network analysis (WGCNA)

Functional annotation of turnip CDS sequences using EggNOG-mapper (version 2.1, http://eggnog5.embl.de/#/app/emapper) (Huerta-Cepas et al., 2017) and WGCNA(version 1.69) analysis of successfully annotated genes in transcripts (Zhang & Horvath, 2005) (Mock-control; 100 µM MeJA spray; 100 mM NaCl watering; 100 µM MeJA spray + 100 mM NaCl watering). In the analysis, the parameters were set as follows. an optimal soft threshold (power) value of 7; minModuleSize of 30, and mergeCutHeight of 0.25. Finally, all co-expression networks associated with *BrrLOXs* were extracted, and the edges with weights below 0.2 were filtered out. We visualized the network connections using Cytoscape software (version 3.8.0, https://cytoscape.org/) (Shannon et al., 2003). Further, the functional annotation of *BrrLOXs* and co-expressed genes was performed using Gene Ontology (GO) and Kyoto Encyclopedia of Genes and Genomes (KEGG) using ggplot2 to identify the top 10 GO annotations and the top 20 KEGG functional annotations (Kanehisa et al., 2016).

## Results

### Identification of turnip *LOX* gene family members

**Figure 1:**
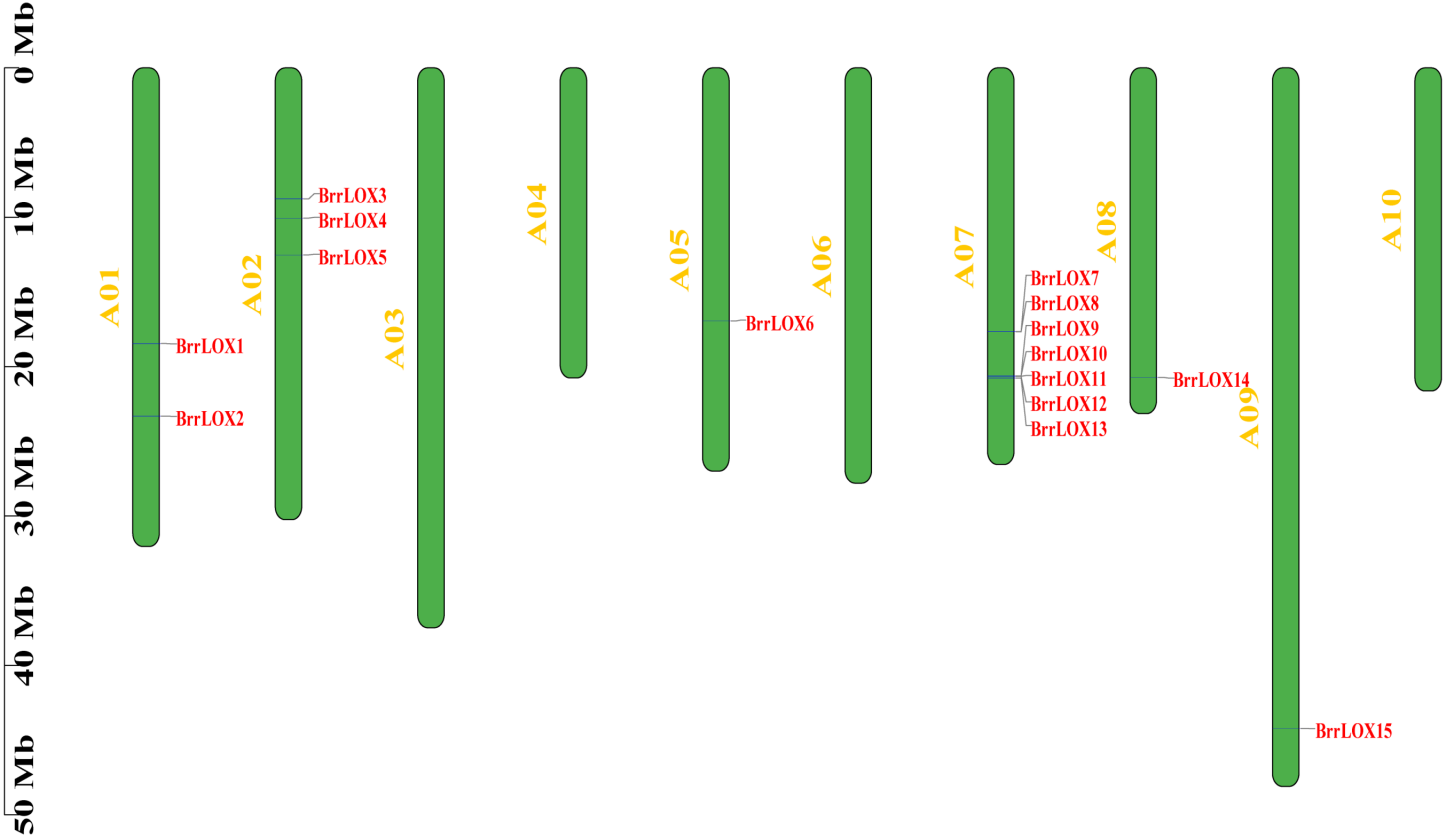
Chromosome localization of *BrrLOX* genes.

A total of 15 *BrrLOX* candidate genes were identified in the turnip genome (Table 1, Table S3) and named sequentially from *BrrLOX1* to *BrrLOX15* based on their chromosomal location. The distribution of *BrrLOX* genes on the chromosomes is shown in Fig. 1. The *BrrLOX* genes were distributed on 6 out of the 10 turnip chromosomes, of which A07 contained seven *LOX* genes, A02 contained three, A01 contained two, and A05, A08, and A09 contained only one each.

**Table 1:** Information of *LOX* gene family in turnip.

**Gene name**	**Gene ID**	**Chromosome location**	**CDS/bp**	**Size/aa**	**Molecular** **weight/kD**	**PI**	**Chloroplast transit** **peptides**	**Subcellular** **localization**
*BrrLOX1*	A01p27670.1_BraTUA	chr01:18452597-18458178 ( +)	2,574	857	97.55	5.18	–	Cytoplasm
*BrrLOX2*	A01p33650.1_BraTUA	chr01:23308367-23312630 ( +)	2,499	832	94.97	6.12	–	Cytoplasm
*BrrLOX3*	A02p16520.1_BraTUA	chr02:8763241-8766727 ( +)	2,703	900	102.92	5.74	Yes	Chloroplast
*BrrLOX4*	A02p18430.1_BraTUA	chr02:10053798-10060145 ( +)	2,742	913	104.28	7.37	Yes	Chloroplast
*BrrLOX5*	A02p22030.1_BraTUA	chr02:12517721-12521356 ( +)	2,760	919	103.87	6.57	–	Cytoplasm
*BrrLOX6*	A05p23650.1_BraTUA	chr05:16936753-16942334 ( −)	2,499	832	94.98	6.06	Yes	Cytoplasm
*BrrLOX7*	A07p26570.1_BraTUA	chr07:17655268-17659036 ( −)	2,715	904	103.10	5.53	Yes	Chloroplast
*BrrLOX8*	A07p26590.1_BraTUA	chr07:17673223-17673580 ( −)	2,715	904	103.10	5.53	Yes	Chloroplast
*BrrLOX9*	A07p32380.1_BraTUA	chr07:20618987-20624363 ( +)	2,676	891	101.59	5.59	Yes	Cytoplasm
*BrrLOX10*	A07p32390.1_BraTUA	chr07:20631218-20636163 ( +)	2,598	865	98.50	5.26	Yes	Chloroplast
*BrrLOX11*	A07p32400.1_BraTUA	chr07:20641143-20646177 ( +)	2,679	892	101.41	5.29	Yes	Chloroplast
*BrrLOX12*	A07p32410.1_BraTUA	chr07:20652307-20656259 ( +)	2,700	899	102.4	5.61	Yes	Chloroplast
*BrrLOX13*	A07p32610.1_BraTUA	chr07:20757766-20762464 ( −)	2,757	918	104.76	7.9	Yes	Chloroplast
*BrrLOX14*	A08p30720.1_BraTUA	chr08:20717446-20721543 ( −)	2,760	919	103.69	6.33	Yes	Cytoplasm
*BrrLOX15*	A09p62970.1_BraTUA	chr09:44213441-44218856 ( −)	2,760	919	103.85	7.16	Yes	Chloroplast

**Figure 2:**
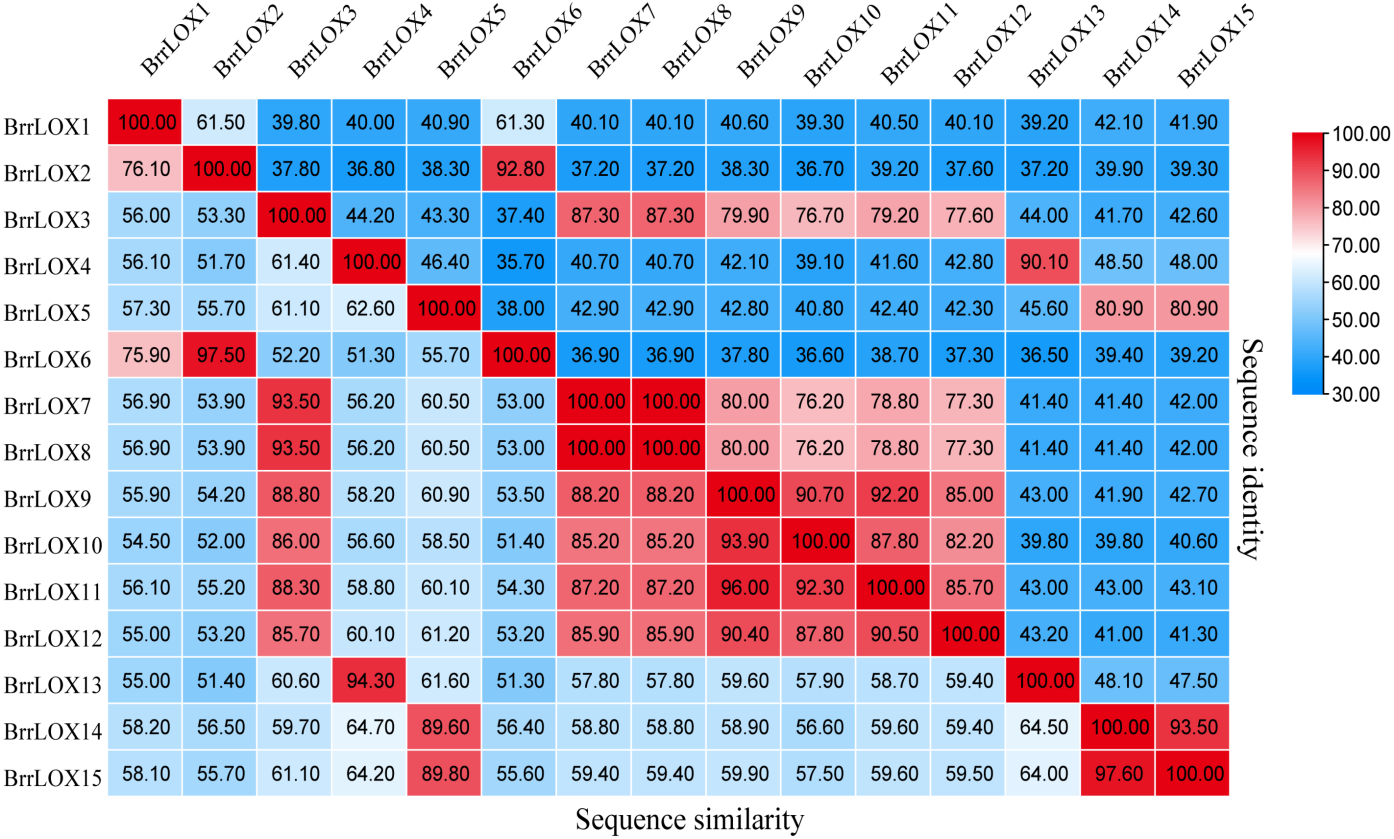
Turnip *LOX* protein Sequence similarity and identity (%). The high, medium and low similarity and identity of *LOX* genes are shown in red, white and blue, respectively.

The CDS lengths of the 15 predicted *BrrLOX* family genes ranged from 2,499 to 2760 bp, respectively, and encoded BrrLOXs proteins from 832 to 919 amino acids in length. The predicted molecular weight of BrrLOX proteins ranged from 94.97 to 104.76 kDa, with a theoretical isoelectric point range of 5.18 to 7.9. The chloroplast transit peptide is present in 11 members of BrrLOXs. Subcellular localisation predicted by WoLF PSORT shows that nine of the BrrLOXs proteins are localised in the chloroplast and the remaining six are localised in the cytoplasm. The protein sequence alignment revealed a sequence similarity of 51.30%–100% and a sequence identity of 35.70%–100% among the *BrrLOX* members (Fig. 2). Specifically, *BrrLOX7* and BrrLOX8 showed the highest similarity and identity (100%). The lowest similarity (51.3%) was between *BrrLOX4* and *BrrLOX6*, *BrrLOX6* and *BrrLOX13*; *BrrLOX4* and *BrrLOX6* showed the lowest identity (35.70%).

### Phylogenetic relationship of LOX family members in different species

**Figure 3:**
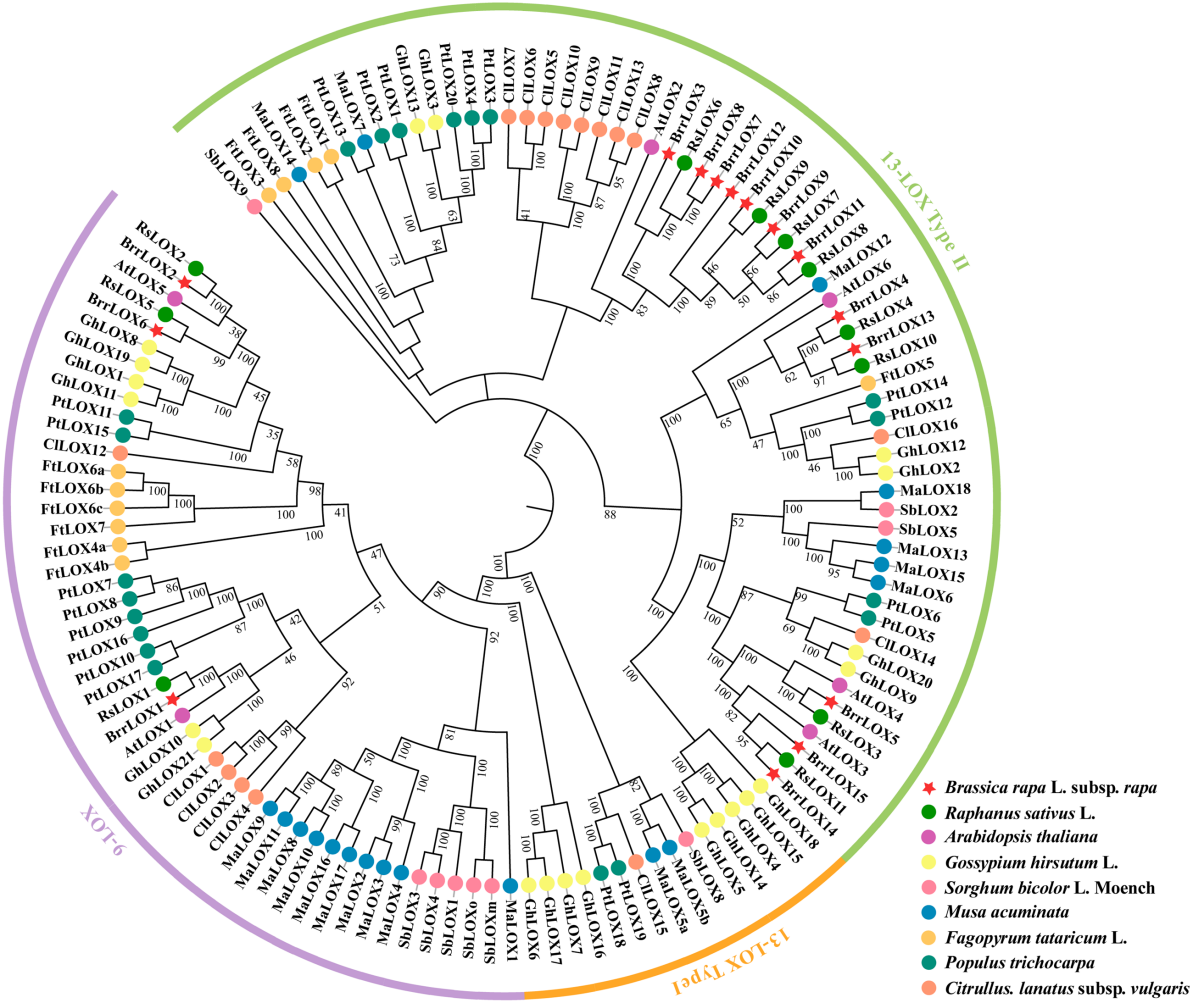
Phylogenetic relationships of LOX proteins of turnip with other plant species. The phylogenetic tree was constructed using the neighbor-joining method with 1,000 bootstrap repetitions. The 9-LOX branch is shown in purple, the 13-LOX type I branch is shown in orange, and the 13-LOX type II branch is shown in green.

We constructed phylogenetic trees based on 128 LOX protein sequences from 9 different species, including turnip (*Brassica rapa* L. subsp. *Rapa*), radish (*Raphanus sativus* L.), Arabidopsis (*Arabidopsis thaliana*), cotton (*Gossypium hirsutum* L.), sorghum (*Sorghum bicolor* L.*Moench*), banana (*Musa acuminata*), buckwheat (*Fagopyrum tataricum* L.), poplar (*Populus trichocarpa*) and watermelon (*Citrullus. lanatus* subsp. *vulgaris*), using the neighbor-joining algorithm in MEGA X software (Fig. 3 and Table S4) (Kumar et al., 2018). The LOX protein sequences were classified into 9-LOX, 13-LOX type I, and 13-LOX type II. Of the 15 BrrLOXs identified, BrrLOX1, BrrLOX2, and BrrLOX6 belong to the 9-LOX subfamily, while the other 12 to 13-LOX type II. The *BrrLOX* gene family analyzed lacked the 13-LOX type I, a result also found in *Arabidopsis thaliana* and other *Brassica species* (*Brassica rapa*, *Brassica oleracea* and *Raphanus sativus*) (Wang et al., 2019).

### Conserved motifs and gene structure of turnip LOXs

**Figure 4:**
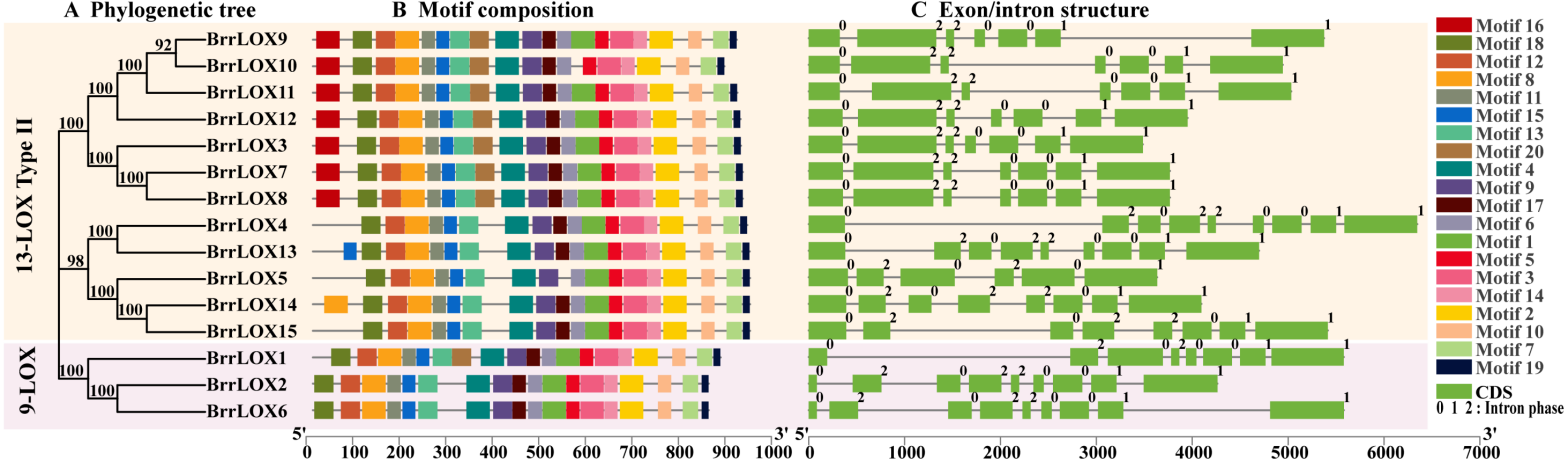
Phylogenetic analysis (A), motif composition (B), gene structure (C), of *BrrLOX* genes. Numbers (0, 1, and 2) indicate intron phases.

The protein motifs of the turnip BrrLOX proteins were analyzed using MEME suite. The analysis revealed 16 conserved motifs (motif2–15, motif18, motif19) widely present in all BrrLOX proteins (Supplementary Figure_S1). The BrrLOXs showed some degree of specificity in motif distribution, with all LOXs except BrrLOX5 containing motif17 (Fig. 4B). Meanwhile, all LOXs except BrrLOX10 have motif1, and motif1 contains a histidine-rich (His) 38 amino acid residue motif consisting of His-(X)4-His-(X)4-His-(X)17-His-(X)8-His. In addition, BrrLOX13 has an additional motif15 at the N-terminus, and BrrLOX14 has an additional motif8 at the N-terminus. These differences in motif arrangement may be responsible for the functional differentiation of the turnip LOX proteins. Our analysis of the gene structure showed that the BrrLOX genes consist of 5–8 introns, with BrrLOX5 having the fewest introns (five) and BrrLOX2, BrrLOX4, BrrLOX6, and BrrLOX13 having the most (eight) (Fig. 4C).

### Gene duplication and collinearity analysis of the *BrrLOX* genes

In the evolution of plants, gene duplication regulates the acquisition of new genes and the creation of new genetic traits (Zhang, 2003). The present study explored the gene duplication events of the LOX family of turnips using the MCScanX software (Wang et al., 2012). In the *BrrLOX* gene family, seven tandem replication gene pairs (*BrrLOX7*/*BrrLOX8*, *BrrLOX9*/*BrrLOX10*/*BrrLOX11*/*BrrLOX12*) and four whole gene replication (WGD) or fragment replication (SD) gene pairs (*BrrLOX2*/*BrrLOX6*, *BrrLOX4*/*BrrLOX13*, *BrrLOX5*/*BrrLOX14*, *BrrLOX14*/*BrrLOX15*) were identified. Furthermore, Ka and Ks were calculated to estimate the evolutionary selection pressure on the duplicate gene pairs of turnip *LOX* genes.

**Figure 5:**
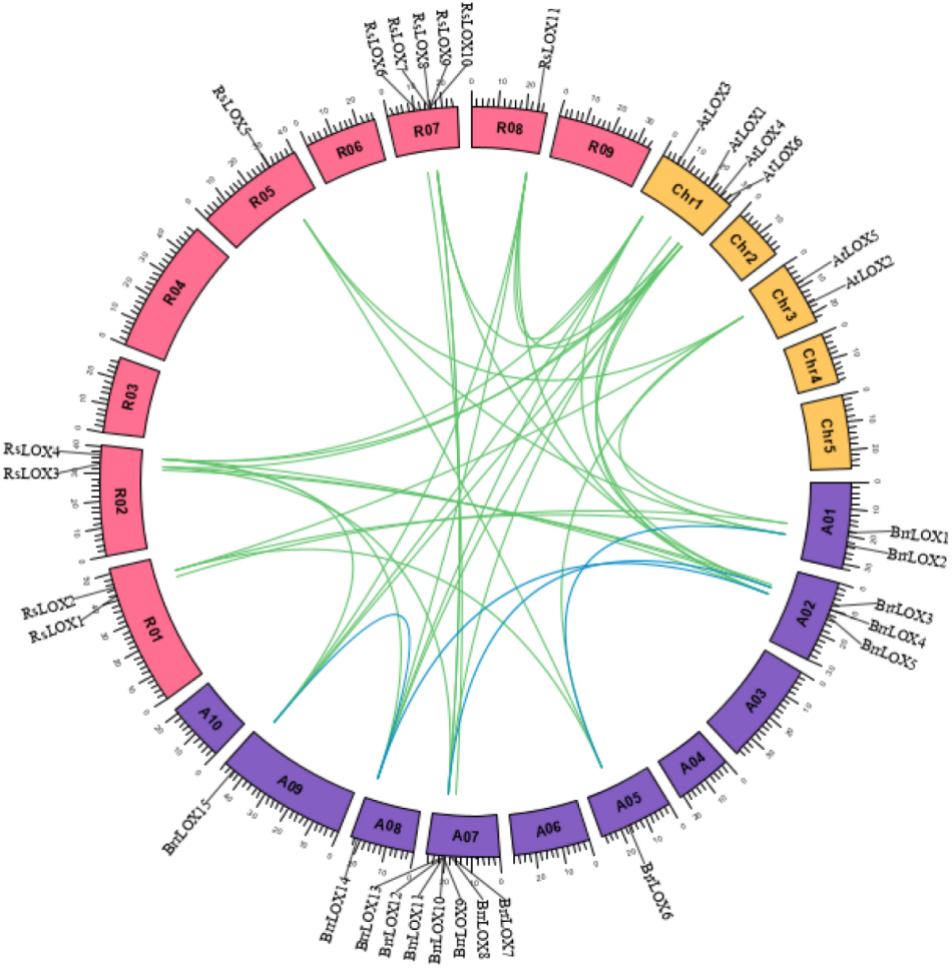
Collinear relationships of Arabidopsis, radish and turnip *LOX* genes. The pink rectangle represents the chromosomes of radish, the yellow rectangle represents the chromosomes of Arabidopsis, and the purple rectangle represents the chromosomes of turnips.The blue lines indicate whole gene duplication (WGD) or fragment duplication (SD) gene pairs of *BrrLOX*, and the green lines indicate *LOX* genes that are homologous between the two species.

The Ka/Ks values for all 11 *LOX* duplicate gene pairs were less than one (Table 2), indicating that *LOX* family genes are mainly influenced by purifying selection during evolution. Analysis of the collinearity of *LOX* genes in Arabidopsis, radish, and turnip by interspecies collinearity (Fig. 5) identified that 8 members of the 15 *BrrLOX* genes were collinear with 5 *AtLOX* genes, giving rise to a total of 11 interspecies collinear gene pairs, mainly on chromosome 2 of turnip. Ten members were collinear with nine *RsLOX* genes (Fig. 5; Table S5), yielding a total of 17 interspecies collinear gene pairs, mainly on chromosome 2. These collinear gene pairs provide a basis for functional studies on the *BrrLOX* genes.

**Table 2:** Estimated Ka/Ks ratios of the duplicated LOX genes in turnip.

**Gene name**	**Gene name**	**Ka**	**Ks**	**Ka/Ks**	**Duplication type**
*BrrLOX7*	*BrrLOX8*	0	0	NA	Tandem duplication
*BrrLOX9*	*BrrLOX10*	0.031829569	0.1236034	0.257513701	Tandem duplication
*BrrLOX9*	*BrrLOX11*	0.039085368	0.140370417	0.278444483	Tandem duplication
*BrrLOX9*	*BrrLOX12*	0.063227762	0.13126891	0.481665936	Tandem duplication
*BrrLOX10*	*BrrLOX11*	0.048024707	0.128237559	0.374497979	Tandem duplication
*BrrLOX10*	*BrrLOX12*	0.066902608	0.117764339	0.568105835	Tandem duplication
*BrrLOX11*	*BrrLOX12*	0.057649265	0.131984388	0.436788515	Tandem duplication
*BrrLOX2*	*BrrLOX6*	0.034172652	0.327625452	0.10430402	WGD or segmental duplication
*BrrLOX4*	*BrrLOX13*	0.047264236	0.380220011	0.124307597	WGD or segmental duplication
*BrrLOX5*	*BrrLOX14*	0.107959478	0.941074251	0.114719405	WGD or segmental duplication
*BrrLOX14*	*BrrLOX15*	0.033123836	0.41651106	0.079526906	WGD or segmental duplication

**Notes.**

Ka non-synonymous substitution rate Ks synonymous substitution rate Ka/Ks the average number of non-synonymous sites

### Analysis of cis-elements in *BrrLOX* gene promoters

**Figure 6:**
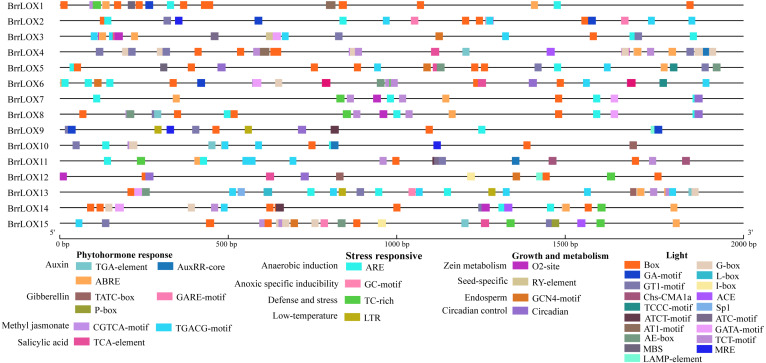
Cis-acting elements identified in *BrrLOX* gene family promoters.

We retrieved a 2,000 bp sequence upstream of the start codon of the *BrrLOX* genes and performed a cis-regulatory element prediction analysis. The analysis identified 38 cis-acting elements classified into four main categories: phytohormone responsive elements, growth and metabolic responsive elements, stress responsive elements, and light responsive elements (Fig. 6). The main phytohormone response elements included TGA-element associated with auxin, GARE-motif, P-box, and TATC-box with gibberellin, ABRE with abscisic acid, CGTCA-motif and TGACG-motif with methyl jasmonate, and TCA-element with salicylic acid, suggesting multiple phytohormones regulate the *BrrLOX* genes. The primary growth and metabolism response elements included circadian, GCN4-motif, Ry-element, and O2-site involved in circadian control, endosperm expression, seed-specific regulation, and zein metabolism regulation, suggesting the roles of *BrrLOX* genes in plant growth and metabolism. The major stress response elements included TC-rich repeats, LTR, ARE, and GC-motif related to defense and stress, low-temperature response, anaerobic induction, and anoxic-specific inducibility. Light response elements, mainly Box 4, G-box, and GT1-motif, exist in all *BrrLOX* promoters regions.

### Weighted gene co-expression network analysis (WGCNA) of *BrrLOXs*

**Figure 7:**
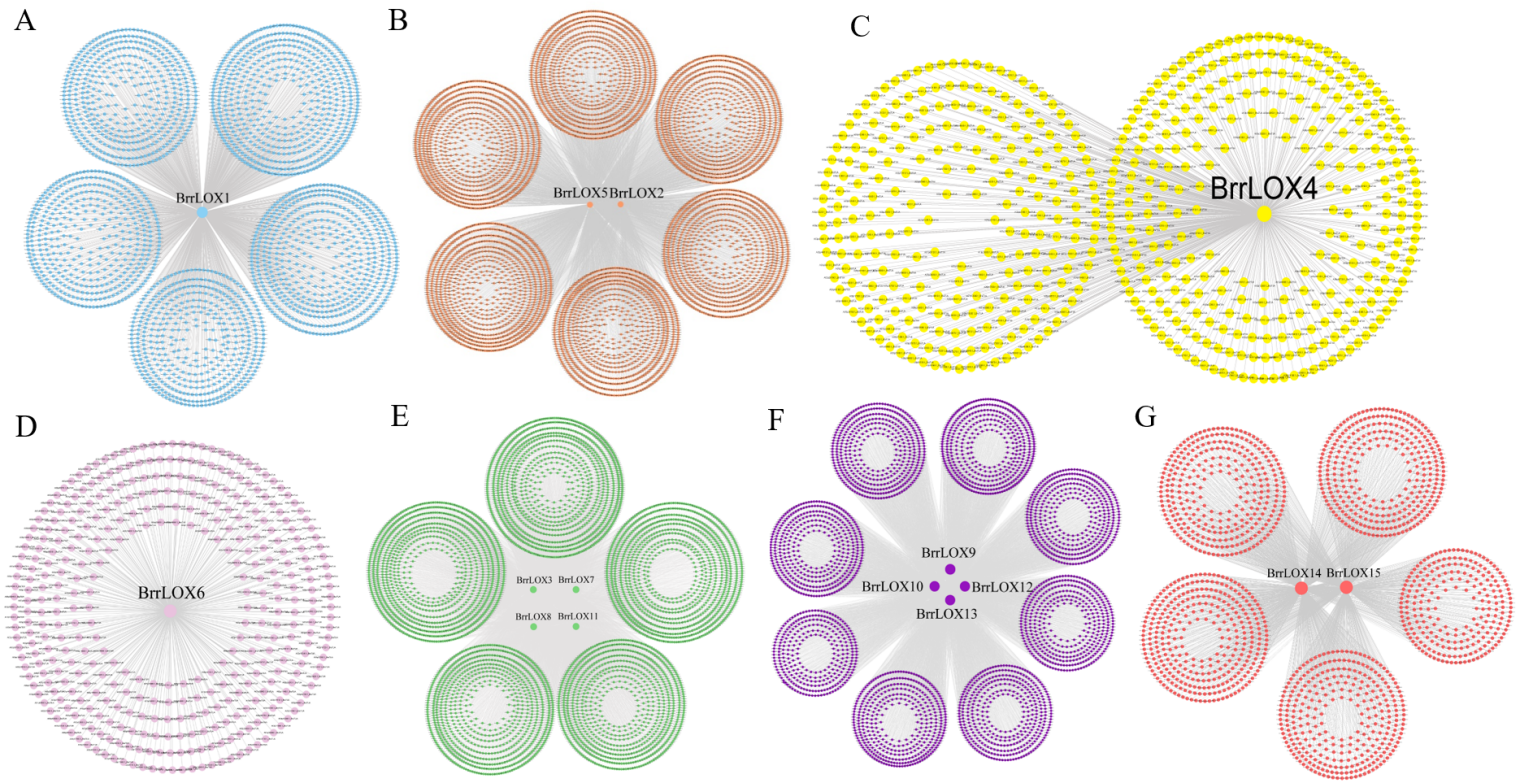
Analysis of *BrrLOX* gene co-expression network. The middle nodes indicate *BrrLOX* genes and all other colored nodes indicate genes co-expressed with *BrrLOX*.

**Figure 8:**
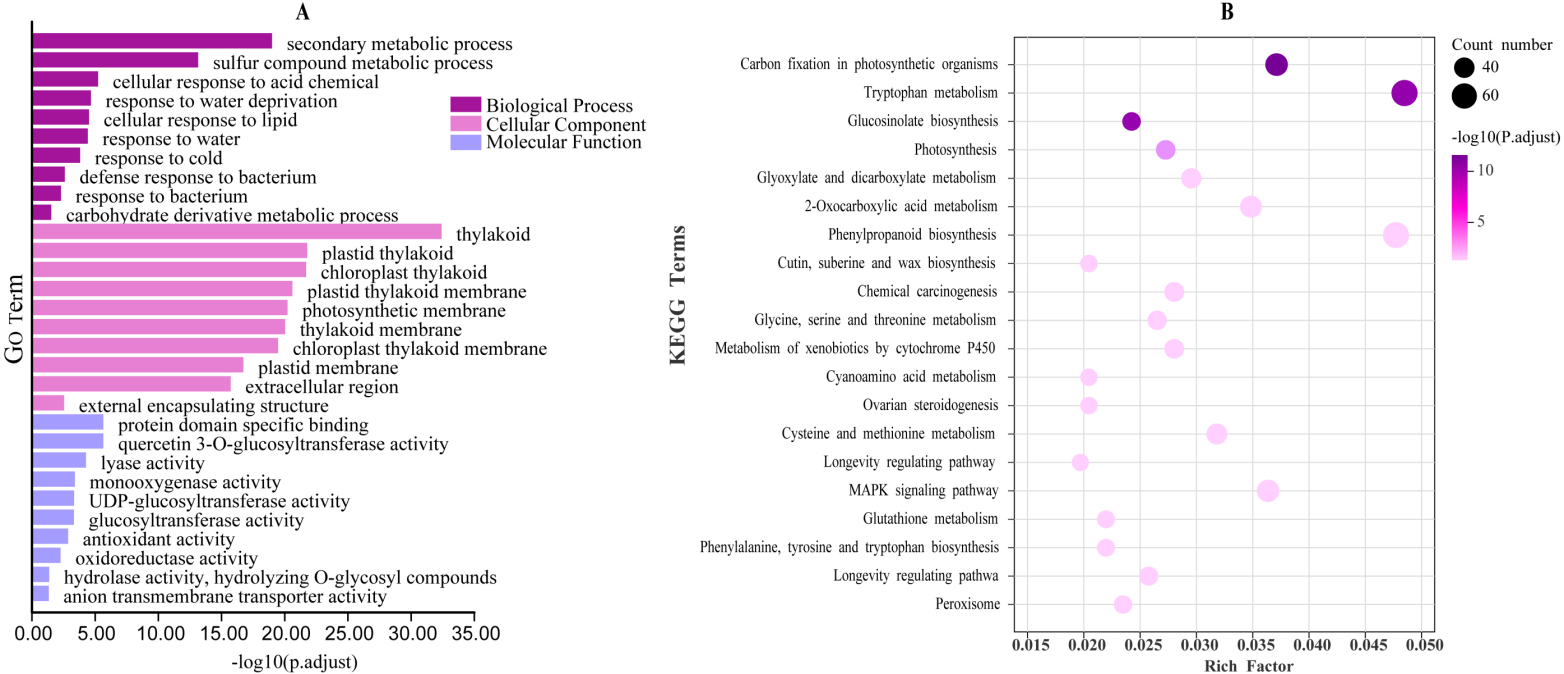
GO and KEGG analyses of genes in the *BrrLOX* gene co-expression network. (A) GO functional annotation; (B) KEGG pathway enrichment.

There were 42,877 functional genes in the turnip CDS sequence that were successfully annotated by EggNOG-mapper (version 2.1) and a co-expression network was constructed using WGCNA based on these functional genes and four transcriptomic data. Co-expression networks associated with *BrrLOX* genes were visualized using Cytoscape software (version 3.8.0), and a total of seven co-expression modules, named modules A to G, were obtained (Fig. 7). The largest of these modules is module B, with 5,426 genes co-expressed with *BrrLOX2* and *BrrLOX5*. The smallest is module D with 383 genes co-expressed with *BrrLOX6*. Furthermore, GO enrichment analysis showed that genes co-expressed with *BrrLOXs* were mainly enriched in the secondary metabolic process, cellular response to lipid, response to a bacterium, sulfur compound metabolic process, and carbohydrate derivative metabolic process in the biological process category (Fig. 8A). Gene annotations were mainly enriched in the cellular components in the thylakoid, extracellular region, plastid thylakoid, chloroplast thylakoid, and plastid membrane. From molecular functions, genes were primarily enriched in tryptophan metabolism, phenylpropanoid biosynthesis, carbon fixation in photosynthetic organisms, MAPK signaling pathway-plant, and 2-oxocarboxylic acid metabolism. According to KEGG functional enrichment analysis, these genes co-expressed with *BrrLOXs* were mainly enriched in tryptophan metabolism, phenylalanine biosynthesis, carbon fixation in photosynthetic organisms, MAPK signaling pathway-plant, and 2-oxocarboxylic acid metabolism (Fig. 8B).

### Transcriptome analysis of the LOX family genes of turnip

**Figure 9:**
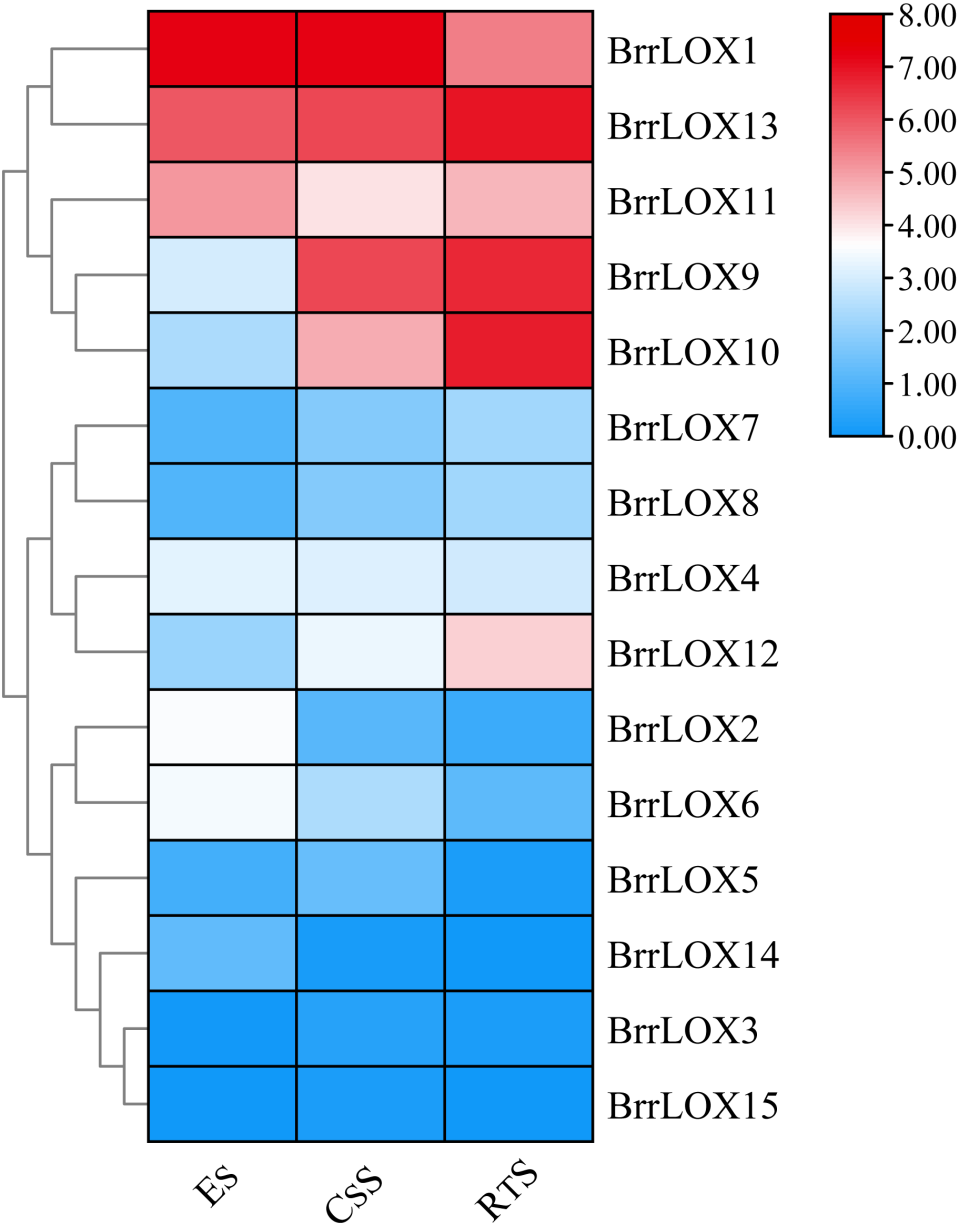
Expression of the *BrrLOX* gene family in the fleshy roots of turnips at different developmental stages. The heat map was created based on publicly available turnip flesh root transcriptome data. In the heat map, the color bars represent the average of the TPM values of log-transformed *BrrLOX* genes in two biological replicate samples. Red represents high expression level, blue represents low expression level. ES, early stage before cortical division; CS, cortical division stage; RTS, secondary root thickening stage.

To investigate the expression of turnip LOX family genes during fleshy roots development, we reanalyzed the expression levels of *BrrLOX* genes using publicly available RNA sequence data from three fleshy roots development stages, including ES (early stage before cortex splitting), CSS (cortex-splitting stage) and RTS (secondary root-thickening stage) (Fig. 9; Table S6), in which *BrrLOX1* and *BrrLOX13* were highly expressed at all three stages; *BrrLOX11* expression was higher in ES and RTS than in CSS, while *BrrLOX9* and *BrrLOX10* expression levels were higher in RTS than in ES and CSS. *BrrLOX12* was highly expressed in RTS. However, *BrrLOX3* and *BrrLOX15* were expressed at 3 periods with little or no expression.

### Expression analysis of *BrrLOX* family genes in turnip under abiotic stress and exogenous hormone treatment

**Figure 10:**
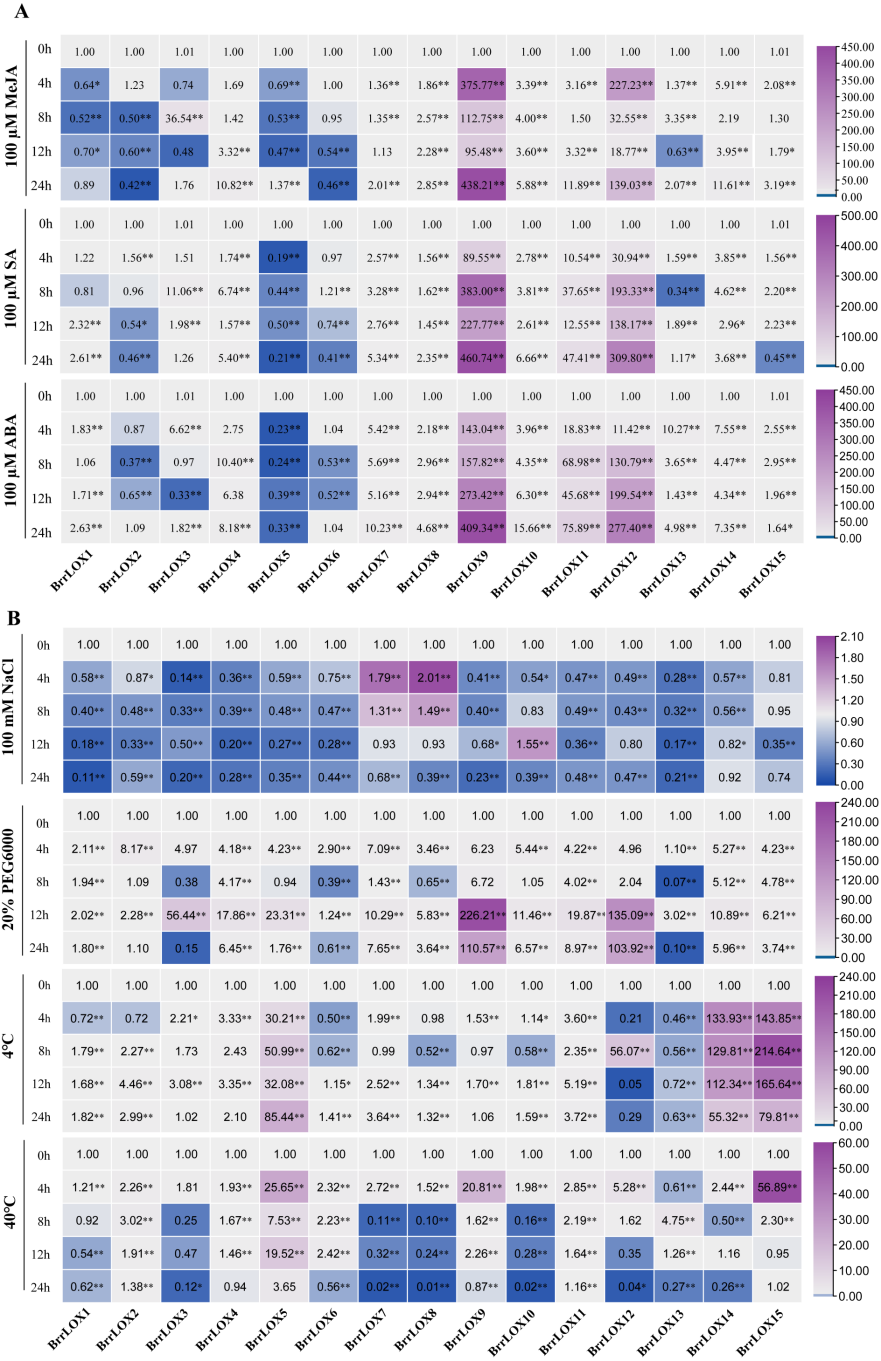
Expression analysis of *BrrLOX* family genes under (A) exogenous hormonal (MeJA, SA, and ABA) and (B) abiotic stress (NaCI, PEG6000, 4 °C and 40 °C) treatments. The relative expression levels of *BrrLOX* genes in turnip leaves were analysed at 4, 8, 12 and 24 h of treatment and compared with their values at 0 h. The gene relative expression was calculated using the 2^−ΔΔCT^ method with *β*-Actin as an internal reference, and values represent mean of three biological replicates. Purple indicates up-regulated expression levels, blue indicates down-regulated expression levels (* *p* < 0.05, ** *p* < 0.01, one-way ANOVA).

We further performed qRT-PCR analysis to detect the changes in the transcript levels of *LOX* genes in turnip leaves under different abiotic stresses (salt stress, drought stress, cold stress, and heat stress) (Fig. 10A and Table S7) and exogenous hormone treatment (MeJA, SA, and ABA) (Fig. 10B and Table S7). Under NaCl treatment, the expression of most genes (*BrrLOX1-6*, *BrrLOX9*, *BrrLOX11-15*) was downregulated, while that of *BrrLOX7* and *BrrLOX8* was upregulated at 4 h and 8 h after exposure; *BrrLOX10* was upregulated at 12 h. Under drought stress, 10 LOX genes were upregulated, among which *BrrLOX9* was most significantly expressed at 12 h. Under low-temperature treatment, *BrrLOX3-5*, *BrrLOX11*, *BrrLOX14*, and *BrrLOX15* genes were upregulated at all time points, with *BrrLOX15* showing a significant high expression at 8 h; *BrrLOX13* was downregulated at all time points. Under high-temperature treatment, *BrrLOX2*, *BrrLOX5*, and *BrrLOX11* were upregulated at all time points, with the most significant expression for *BrrLOX15* at 4 h.

Furthermore, the *BrrLOX* genes showed different expression patterns under various hormonal treatments (Fig. 10B). *BrrLOX1* gene expression was downregulated under the MeJA treatment and upregulated under the ABA treatment. *BrrLOX4*, *BrrLOX7-12*, and *BrrLOX14* were upregulated with three exogenous hormones, with the highest effect for *BrrLOX9* at 24 h.

## Discussion

Oxidized lipid compounds (*e.g.*, jasmonic acid, green leaf aldehydes, etc.) synthesized via the LOX pathway play important roles in plant growth and development and stress responses, such as seed germination (Li et al., 2018), fruit development (Contreras et al., 2017), drought (Sofo et al., 2004), injury (Xu et al., 2003), and pests and diseases (Christensen et al., 2013). However, fewer studies have been reported on the analysis of the functions of members of the turnip *LOX* gene family, therefore, this study identified the LOX family members in turnip based on the turnip reference genome and analyzed their structure and evolution. Further, transcriptome data was analyzed to understand their role during development, and qRT-PCR was used to study their roles in response to abiotic stresses (cold, heat, simulated drought, and salinity) and exogenous hormone treatments (MeJA, ABA, and SA).

The present study identified 15 *BrrLOX* family genes in turnip by BLASTP search and CDD structural domain test, which was more than in Arabidopsis (six) (Umate, 2011) and radish (11) (Wang et al., 2019), but less than in cucumber (23) (Liu, Liu & Jiang, 2011) and cotton (21) (Shaban et al., 2018). These observations revealed that the number of LOXs is not proportional to genome size and suggested that the *LOX* genes are not conserved across dicotyledons and have changed during evolution. Phylogenetic tree analysis divided the turnip LOX family into two subfamilies, 9-LOX (*BrrLOX1*, *BrrLOX2*, *BrrLOX6*) and 13-LOX Type II (*BrrLOX3*, *BrrLOX4-5*, *BrrLOX7-15*), lacking 13-LOX Type I. This result is consistent with the classification of the LOX gene family of other Brassica species (Wang et al., 2019). Typically, 13-LOX could be divided into two subfamilies, 13-LOX Type I and 13-LOX Type II; based on the presence or absence of chloroplast transit peptide, where 13-LOX Type I lacked chloroplast transit peptide and had >75% sequence similarity, while LOX of 13-LOX type II had chloroplast transit peptide and had >35% sequence similarity (Bell & Mullet, 1993). In the present study, LOX belonging to 13-LOX type II, with the exception of BrrLOX5, contained chloroplast transit peptides. Sequence similarity analysis revealed that, with the exception of tandem replication genes, the sequence similarity of the few belonging to 13-LOX type II was still greater than 80%, which was significantly higher than 35%. This finding is not entirely consistent with the above classification, but such results have been found in banana (Liu et al., 2021), so we think it may be related to the structural diversity of *LOX* genes.

Gene duplication is a major mechanism that increases the number of gene family members and functional diversity (Magadum et al., 2013). Therefore, we analyzed the gene duplication events in the turnip genome to understand the formation and functional diversity of the *LOX* gene family. Previous studies identified two tandem repeat gene pairs and four fragment repeat gene pairs in the *LOX* family genes of banana and two tandem repeat gene clusters in watermelon (Liu et al., 2021; Liu et al., 2020). The present study found that four pairs were formed by whole gene duplication (WGD) or fragment duplication (SD), accounting for 46.67% (7/15) of the total number of *LOX* genes, and seven pairs by tandem duplication, accounting for 40.00% (6/15) of the total number of *LOX* genes. Our results suggest that tandem replication and fragment replication are the predominant replication events of the *BrrLOX* genes. In addition, Ka/Ks values for the homologous gene pairs were less than one, indicating that the turnip *LOX* genes have undergone purifying selection, promoting the stability of gene function. Eight of the 15 *BrrLOX* genes showed collinearity with Arabidopsis *LOX* genes and 10 with radish. The presence and identification of these collinear gene pairs will facilitate studies on turnip genes compared with the known functional genes of Arabidopsis and radish.

Weighted gene co-expression network analysis (WGCNA) can cluster genes with similar expression patterns into the same module, and analysis of genes in these modules can provide a better understanding of gene interaction networks and biological functions. Therefore, in our study, a total of seven co-expression modules associated with the *BrrLOX* gene were screened using WGCNA analysis. Most of the neighboring genes in the *BrrLOX* gene co-expression network were associated with sub-metabolic processes, bacterial defense, drought stress, cold stress, and photosynthesis. In addition, the *BrrLOX* promoters contain a large number of cis-acting elements associated with growth and development and stress response. These results suggest that *BrrLOX* genes are extensively involved in the growth and development of turnips and in response to various biotic-abiotic stress pathways.

In plants, LOXs play important roles in regulating the development of various organs, including roots. In Arabidopsis, oxidized lipids produced by the 9-LOX pathway regulate root development by affecting transcriptional changes in cell wall modification genes (Vellosillo et al., 2012). In potato, the antisense expression suppressor mutant *POTLX-1* inhibited LOX activity and root tuber development by inhibiting the expression of *LOX1*-like genes (Kolomiets et al., 2001). In this study, transcriptome data analysis of three fleshy roots developmental stages revealed that *LOX* family genes were differentially expressed in turnip fleshy roots, with three *LOX* genes highly expressed in the ES phase, five in the CSS phase, and six in the RTS phase, suggesting their roles in the development of turnip fleshy roots. Specifically, *BrrLOX11* was more expressed in RTS and ES than in CSS, *BrrLOX9* and *BrrLOX10* in RTS than in CSS and ES phases, implying that different *BrrLOX* members perform different functions at different developmental phases.

Furthermore, the LOX pathway produces secondary metabolites involved in plant responses to various abiotic stresses. In tomato, overexpression of the *TomLOXD* gene increased resistance to high-temperature stress by catalyzing the α-linolenic acid pathway (Hu et al., 2013). The melon *CmLOX10* gene is heterologously overexpressed in Arabidopsis thaliana and improves drought tolerance in transgenic plants by increasing the synthesis of endogenous jasmonic acid (Xing et al., 2020), the pepper *CaLOX1* gene increased tolerance under high salt stress and severe drought stress by regulating lipid peroxidation and various other pathways (Lim et al., 2015). The present study analyzed the expression profiles of *BrrLOX* genes under different stress treatments by qRT-PCR and found altered expression of all genes. Here, 12 *LOX* genes were downregulated under NaCl stress, and only 3 (*BrrLOX7*, *BrrLOX8*, *BrrLOX10*) were upregulated, consistent with the observations in radish under salt stress (Wang et al., 2019). 10 *LOX* genes were expressed higher than the control under drought stress, 6 under low temperature, and 3 under high temperature, indicating the role of *BrrLOX* genes in multiple stress responses and this result was also found in tomato (Upadhyay, Handa & Mattoo, 2019). The present study also revealed that 4 tandemly replicated genes, *BrrLOX9*, *BrrLOX10*, *BrrLOX11* and *BrrLOX12*, had significant differences in gene expression levels although they were structurally similar. We speculate that the turnip LOX gene family has undergone gene subfunctionalization during the evolutionary process, leading to differences in the roles of homologous genes under different stresses.

Several studies have found that exogenous JA/MeJA treatment strongly induces *LOX* gene expression. For example, in watermelon, the expression of *CILOX* genes was significantly up-regulated after exogenous jasmonic acid treatment, with the expression of *CILOX7* being 60-fold higher than that of the control (Liu et al., 2020). In banana, the expression of eight *MaLOX* genes was up-regulated after exogenous MeJA treatment (Liu et al., 2021). Our findings are consistent with these results; here, MeJA upregulated the expression of nine *BrrLOX* genes, with *BrrLOX9* being the most significantly upregulated compared to the control (> 90-fold). In addition, among the nine up-regulated *BrrLOX* genes, only the promoter regions of four *LOX* genes (*BrrLOX10*, *BrrLOX11*, *BrrLOX14*, *BrrLOX15*) contained MeJA cis-acting regulatory elements, and there was no direct correlation between the number of these cis-acting elements and gene expression patterns. Therefore, the potential role of MeJA cis-acting elements in gene regulation needs to be verified in future studies.

ABA is another important plant stress hormone. A recent study showed that the melon *CMLOX13* gene expression in Arabidopsis enhanced tolerance to severe drought by regulating ABA accumulation and stomatal closure (Xing et al., 2019). In the present study, nine *BrrLOX* (*BrrLOX1*, *BrrLOX4*, *BrrLOX7*, *BrrLOX9-12*, *BrrLOX14*, *BrrLOX15*) genes were up-regulated in expression under ABA and drought stress treatments, among which, *BrrLOX9* had the highest relative expression levels under both stresses, therefore, in the next study, we will select *BrrLOX9* as a candidate gene to explore the role of *BrrLOX* gene in drought stress. In addition, we identified defense and stress response elements, low temperature response elements and light response elements in the promoter region of the *LOX* genes of the turnip. These results suggest that *LOX* genes may be involved in a variety of biological processes during the growth and development of turnips.

## Conclusions

The present study identified 15 *LOX* genes in the turnip genome with intact structural domains of lipoxygenases, which were unevenly distributed on six chromosomes of the turnip genome. Phylogenetic analysis classified turnip *LOX* genes into two subgroups, 9-LOX and 13-LOX type II. Gene structure analysis indicates that *BrrLOX* genes are highly conserved in the same subfamily or evolutionary branch, which may indicate that they have similar biological functions. Gene replication analysis indicates that tandem and fragment replication play a dominant role in the expansion of the *BrrLOX* family and the generation of new *BrrLOX* genes. Further qRT-PCR showed that different *LOX* genes responded to abiotic stresses, such as salt, drought, low and high temperature, suggesting their roles in abiotic stress tolerance. The WGCNA constructs suggest that *BrrLOX* may be involved in plant growth and development, and biotic and abiotic stress responses. Thus, our results will help to investigate further the key functions of *LOX* genes in evolution, root development, and abiotic stress tolerance.

##  Supplemental Information

10.7717/peerj.13746/supp-1 Supplemental Information 1 Motif 1-20 sequences

10.7717/peerj.13746/supp-2 Supplemental Information 2 Expression matrix of transcriptome data

10.7717/peerj.13746/supp-3 Supplemental Information 3 RT-PCR primers of BrrLOXs

10.7717/peerj.13746/supp-4 Supplemental Information 4 CDS sequences of predicted candidate genes for *BrrLOXs*

10.7717/peerj.13746/supp-5 Supplemental Information 5 Plant LOX protein sequences used for phylogenetic tree construction

10.7717/peerj.13746/supp-6 Supplemental Information 6 Interspecies and intraspecies co-linear gene pairs

10.7717/peerj.13746/supp-7 Supplemental Information 7 TPM values of RNA-seq data in different tissues of turnip

10.7717/peerj.13746/supp-8 Supplemental Information 8 qRT-PCR expression analysis data under abiotic stress

## Additional Information and Declarations

### Data Availability

The following information was supplied regarding data availability: The raw data are available in the Supplementary File.
