# Supplementary figures and images for: Genome-wide identification and expression pattern analysis of lipoxygenase gene family in turnip (Brassica rapa L. subsp. rapa)
Source: PeerJ. 2022 Jul 22;10:e13746. doi: 10.7717/peerj.13746 (PMC9310782; doi:10.7717/peerj.13746)

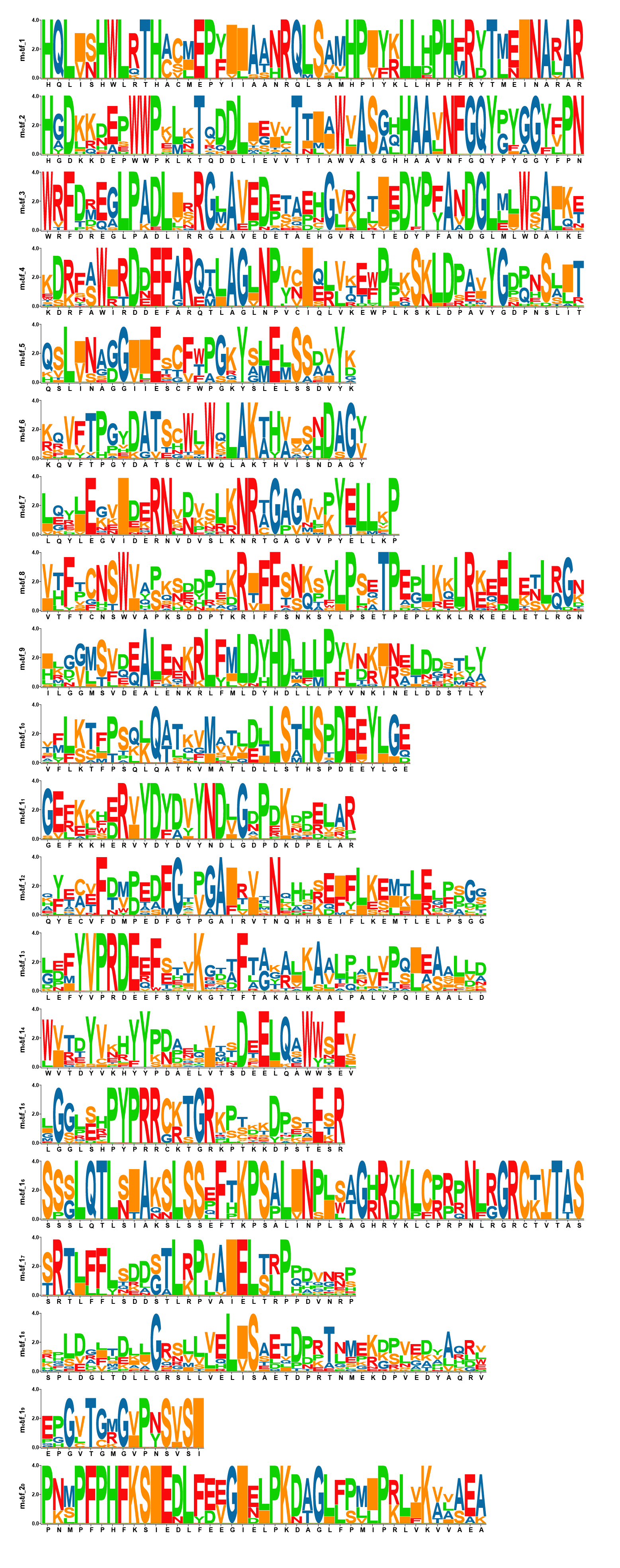

Supplement: Supplemental Information 1 [file peerj-10-13746-s001.png]
